# System Proposal for Mass Transit Service Quality Control Based on GPS Data

**DOI:** 10.3390/s17061412

**Published:** 2017-06-16

**Authors:** Gabino Padrón, Teresa Cristóbal, Francisco Alayón, Alexis Quesada-Arencibia, Carmelo R. García

**Affiliations:** Institute for Cybernetics, Campus de Tafira, Las Palmas de Gran Canaria, University of Las Palmas de Gran Canaria, Las Palmas 35017, Spain; gabino.padron@ulpgc.es (G.P.); teresa.cristobalb@gmail.com (T.C.); francisco.alayon@ulpgc.es (F.A.)

**Keywords:** intelligent transport systems, public transport systems, operation control, automatic vehicle location

## Abstract

Quality is an essential aspect of public transport. In the case of regular public passenger transport by road, punctuality and regularity are criteria used to assess quality of service. Calculating metrics related to these criteria continuously over time and comprehensively across the entire transport network requires the handling of large amounts of data. This article describes a system for continuously and comprehensively monitoring punctuality and regularity. The system uses location data acquired continuously in the vehicles and automatically transferred for analysis. These data are processed intelligently by elements that are commonly used by transport operators: GPS-based tracking system, onboard computer and wireless networks for mobile data communications. The system was tested on a transport company, for which we measured the punctuality of one of the routes that it operates; the results are presented in this article.

## 1. Introduction

In modern societies, mobility plays a significant role in quality of life and is an aspect that has a considerable influence on the socioeconomic development of people. Because of this, we are currently witnessing a large-scale use of transport systems that is giving rise to problems such as environmental degradation, traffic congestion and an increased risk of road accidents. Different local, national and international agencies are constantly producing reports that highlight both the importance of transport systems and the need to address the problems inherent therein. It is estimated that over 40% of the world population spends at least an hour a day travelling by road [1]; the relationship between the rising number of people with heart, respiratory and brain diseases and high levels of pollution has been proven by medical studies [2]; the World Health Organisation [3] estimates that around 3 million people die annually due to outdoor air pollution; and finally, according to statistics, every year 250,000 people in the European Union [4] are seriously injured as a result of traffic accidents, with road fatalities at about 10% of that figure. Transport agencies are tackling these mobility-related problems mainly by, firstly, promulgating traffic rules and regulations that benefit transport and, secondly, using technology to develop more efficient transport systems. A highlight of the latter approach is the use of intelligent transport systems (ITS) to develop public transport systems that are more efficient, environmentally friendly and attract citizens away from the use of private transport. The key element in making a public transport system attractive to citizens is quality, an aspect that means responding to mobility needs and running on schedule. There is general consensus on the aspects that influence public transport users’ perception of service quality. These are, mainly, punctuality, real-time availability of information on timetables including incidents affecting the service, and shorter waiting times at stops. There are even recommendations and standards, and legislation such as that which exists in the European Union [5], which sets out the parameters to be considered when assessing the quality of service provided by a public transport operator, which include timetable adherence. Because public road passenger transport is affected by external variables such as traffic and weather conditions, user demand, etc., comprehensive quality control in a transport network requires continuous assessment. And this requires elements from the transport network infrastructure, such as sensors, and computer and communications systems, to obtain the required data, which are on a massive scale in the case of medium to large transport networks.

This article presents a system for continuously and systematically evaluating the service quality of a public road passenger transport operator. This systematic evaluation is performed using vehicle tracking systems and mobile communications infrastructures used by transport operators, the set-up of which does not require any specific deployment of new devices. In addition, the data provided by the proposed system are integrated into the data model used by the transport operator, thus allowing detailed and continuous analysis of important aspects such as the punctuality or regularity of the services provided to users.

In addition to this introductory section, this article is organised into seven sections. The second section reviews related studies, focusing on the criteria, parameters and systems used to measure the quality of service in public transport by road. The third section explains a number of preliminary ideas and concepts to be used in the rest of the article: a general description of the positioning system used by the system (GPS), and a presentation of the conceptual and formal model of the entities involved in evaluating quality of service, as well as the criteria and parameters used. Having reviewed related studies, introduced the proposed system and formalised the problem, in the fourth section we explain the challenges that must be tackled in quality control of a public road passenger transport service. The fifth section describes the proposed system, looking specifically at its operating principles and its constituent components; the system was tested under real conditions in a local transport company on the island of Gran Canaria. In the sixth section the results obtained when evaluating the quality of service are discussed, based on the criterion of punctuality on one of the company’s routes for a period of one year. Finally, the seventh section contains the main conclusions.

## 2. Related Studies

In this section we shall review the studies published about quality of service in regular road passenger transport, with particular reference to publications that deal with criteria and metrics associated with, and systems used to obtain the data required for, quality control. Peeks et al. [6] ranked the factors that influence public transport users’ perception of quality according to different levels of importance. On the first level is safety and reliability, on the second level, the speed of the journey, and on the third level, convenience, comfort and finally, the experience. According to Van Oort [7], three factors have a particularly negative effect on the quality of public transport: unforeseen increases in waiting times for travellers, the time spent by the traveller in situations of overcrowding due to an overloaded public transport network, and delays in arrival times at destinations due to the variability of travel times. From the point of view of the traveller, the first two factors influence the feeling of comfort offered by the public transport service and the third factor influences their convenience-based decision on whether to use public or private transport. Moreira-Matias et al. [8] carried out an exhaustive review of the various methods that have been used to improve and optimise public passenger transport, which included the evaluation of adherence to public transport timetables. Timetable adherence means arriving at stops and stations on time, a key aspect in the user’s perception of public transport service quality. It is therefore essential that timetables are created using techniques that schedule departure and arrival times as accurately as possible. In this vein, the problems in predicting public transport bus arrival times at stops have been addressed using different techniques. For example, Yu et al. [9] used Kalman filters, Chang et al. [10] proposed a solution based on non-parametric regressions, and Jeong et al. [11] used neural networks. Once the route timetables have been established, various criteria and associated metrics have been used to evaluate adherence to those timetables. Turnquist [12] identified two strategies for planning regular road passenger transport routes: the first consists in planning routes by frequency, and the second, by timetable. The first planning strategy is used for urban transport and the criterion applied is regularity. The second planning strategy is used for long-distance transport and the criterion applied is punctuality. A starting point of reference is the study by Polus [13], in which he proposed the following indicators for arterial routes: overall travel time, congestion index, overall travel speed and overall delay. Subsequently, and making use of technological advances in onboard systems that enable a larger volume of data to be recorded, timetable adherence has been analysed at a far greater level of detail in the transport network: at a single stop, or on a given section of the route. Nakanishi [14], Strahman et al. [15] and Barabino et al. [16] proposed using the following parameters: on-time performance (OTP), run time variation (RTV), headway variation (HV) and excess waiting time (EWT). The first two are used to measure punctuality and the last two, to measure regularity. Lin et al. [17] proposed another indicator, called adjacent site punctuality rate. To obtain all these indicators it is necessary to gather data on vehicle location and the times that the vehicles pass through each of the control points on the route. Continuous monitoring of all the stops on the route gives rise to the challenge of managing a massive amount of data, more so when the transport network contains a considerable number of stops, which is the case of medium to large metropolitan areas.

According to Furth et al. [18], the data used to check and improve public passenger transport planning may be obtained through three different data sources: surveys of public transport users, and automatic passenger counter (APC) and automatic vehicle location (AVL) systems. The AVL systems provide the data source for vehicle location and times of arrival and departure at stops. Several positioning technologies are used by AVL systems. According to Riter et al. [19], they can be broken down into three categories: radiolocation systems that establish position with radio signal triangulation techniques, estimation of the direction and distance travelled (dead reckoning) or proximity detection systems. Currently, radiolocation systems are the most used, particularly the Global Positioning System (GPS) [20], although proximity detection systems are also used, such as the system proposed by Zhou et al. [21], who used a Radio Frequency Identification (RFID) system to detect the presence of the vehicle at a stop in order to assist travellers with special needs. Examples of AVL system proposals using GPS have been put forward by Mazlooumi et al. [22], who used GPS data to analyse public transport travel time variability; Zhao et al. [23], who used GPS data to determine optimal slack time for planning schedule-based services; Derevitskiy et al. [24] studied traffic conditions in the transport network using GPS data; Cortes et al. [25] used the same data to estimate bus speed; García-Castro et al. [26] used GPS data from private vehicles to calibrate the input data used in different techniques for modelling road traffic and the effects that it causes. GPS is a case of a global navigation satellite system (GNSS), which has become popular due to its features and the low cost of GPS receivers. In recent years navigation systems have been used that are able to use the signal from several different constellations of satellites to obtain more precise position measurements than those obtained using only signals from GPS satellites; along these lines, Dabove et al. [27] studied the use of the GLONASS constellation.

The system described in this article evaluates adherence to scheduled passing times on a public road passenger transport network. The system autonomously and continuously records all the events that describe the activity carried out by the transport vehicle (bus) by referencing them in space and time with a level of granularity that enables reliability evaluations using the various indicators mentioned above. The purpose of this may be to adapt to different modes of transport, such as long-distance transport routes on which timetable adherence at stops is a key element in the quality of service, or urban transport, where quality is associated with vehicle frequency. Our proposed system uses an AVL system that periodically records the vehicle position data every time the GPS reading is taken, integrating these data into the model used to represent the transport network, the planning of services and the activities carried out by the transport company.

## 3. Preliminary

### 3.1. Global Positioning System (GPS)

The positioning system used in our proposal is GPS. This system allows the location of mobile objects (airplanes, ships, cars, etc.) to be determined, thus enabling their route to be monitored. To provide geolocation information, GPS uses a constellation of satellites that emit a signal that is received and processed by the receivers built into mobile devices. When a GPS receiver is able to receive signals from at least four different satellites, a three-dimensional geolocation is obtained (latitude, longitude and altitude). In addition to position data, the GPS receiver also provides the time that the data were received; this information comes from the atomic clock on the GPS satellite, which is encoded and sent with the signal emitted by the satellite. The receiver also provides data indicating how many satellites were used to obtain the data (data quality) or the time elapsed since they were obtained (data age). In a conventional GPS receiver, position data errors vary depending on various factors, with errors of up to 100 m in the early days of civilian use, when the intentional degradation named Selective Availability (SA) was active [28]. There are positioning systems based on GPS that provide very precise measurements, such as Differential GPS, which achieves accuracy of less than 2 m. The errors in GPS data are due to different causes. A first source of error is found in the GPS system itself: in the errors that occur in the satellite orbits, caused by drift from true time by the atomic clocks on the satellites and by the geometric configurations of the satellites “visible” to the GPS receiver. Since its inception, GPS performance has gradually improved; Table 1 presents some performance metrics for the system from a study conducted in 2013 by the University of Texas, Austin [29]. Among other performance metrics analysed by this study, this table shows the metrics related to Signal-in-Space (SIS), User Range Error (URE), Accuracy, Position Service Availability, and Position Accuracy. AOD refers to Age of Data, which is provided for each GPS reading and indicates the time elapsed since the last position was calculated.

**Figure 1:**
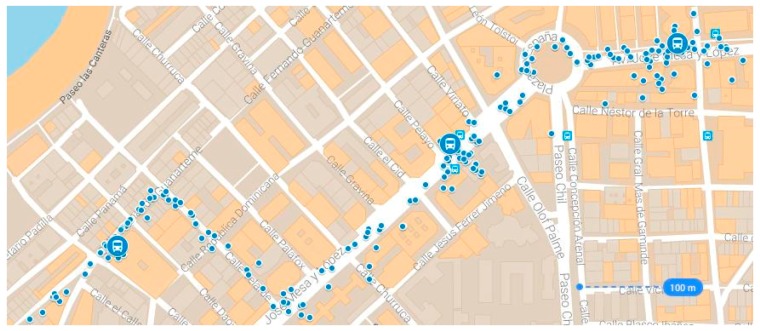
Examples of GPS location data for public transport buses on a fixed route. The positions are represented by the blue dots.

A second cause of error is the propagation medium, i.e., refraction in the ionosphere and troposphere, and errors produced by the same signal reaching the receiver by two or more different paths because it has bounced off buildings or natural elements; this error is called multipath error. A final cause of error in position data is due to errors in the GPS receivers. Our proposed system uses conventional GPS receivers installed in vehicles, and the accuracy of the data that they provide depends on the satellite constellation that they are able to view at the time that the position and geometry of the constellation is calculated. In an ideal scenario, with constellations of more than 7 satellites an accuracy of less than 2 m can be obtained. Given the different sources of error in the GPS coordinates, in the case of public transport vehicles, multipath errors are particularly relevant, especially when the vehicles run through urban areas. Considering the above explanation of GPS, the user accuracy depends on a combination of satellite geometry, URE, and local factors such as signal blockage, atmospheric conditions, and receiver design features/quality, adopting our system a maximum possible error in position data of 25 m, taking into account that the SIS URE Accuracy is behind 13 m during normal operations at any AOD and the metioned external factors (atmospheric conditions, multipath, technical manufacturer specifications, …); henceforth this maximum error will be represented by *E*. Figure 1 is a map illustrating the position data captured by public transport buses that follow a route in an urban area. The blue dots represent the position data, and we can see that some of these coordinates stray from the road because of GPS data errors.

It is often the case that, once the vehicle’s GPS geolocation has been obtained, it has to be positioned on the transport network. This requires a geographic database that contains the positions of the different points on the transport network. To locate a vehicle on the network, the distance between its GPS position and the position of the different points on the transport network is used. This distance can be calculated using different formulae, all of which result in errors since the Earth is not a perfect shape; it is neither a perfect sphere nor a perfect ellipsoid. In general, the distance formula with the lowest error is the haversine formula [30], and is therefore used by the proposed system. Given two points, *p*1 and *p*2, with coordinates expressed in radians (*lat*_1_, *lon*_1_) and (*lat*_2_, *lon*_2_), the value of this distance is calculated using the following expression:

$$Dist\left(p1,p2\right)=2\times R\times \arcsin(\sqrt{A}),$$

where *R* is the radius of the Earth at the equator and *A*, a factor calculated as follows:

$$A=\sin^{2}\left(\frac{\Delta lat}{2}\right)+\cos(lat_{1})\times \cos\left(lat_{2}\right)\times \sin^{2}\left(\frac{\Delta lon}{2}\right),$$

$$\Delta lon=lon_{2}-lon_{1},$$

$$\Delta lat=lat_{2}-lat_{1}.$$

This distance function produces an error, owing to the fact that the Earth is not a perfect sphere and the radius of curvature is not always the same. The maximum error occurs when the points are located at the greatest possible distance, i.e., in the antipodes—a distance of approximately 20,000 km—the maximum error being 2 km.

### 3.2. Conceptual and Formal Model of the Public Transport Network and Activity

This section will describe all the concepts involved in evaluating service reliability. These concepts are derived from international standards for conceptual transport data models; specifically, Transmodel [31], a European reference data model for public transport information. The first concept that we took from this conceptual model is that of transport network. According to this model, a transport network is made up of all the entities that represent the places, roads and routes necessary to spatially describe vehicle operations. One such entity is points on the transport network. These points may represent places where various actions are performed; for example: point where passengers board or alight from the vehicle, timetable control points, points of interest, etc. Each of these activities is associated with a classification, and a point may belong to more than one classification. For example, it may be both a point where passengers board and alight and a timetable control point. The points of particular interest for the purposes of our system are those points where passengers board or alight from the vehicle, which will be called, generically, stops. Examples of these points are platforms at stations or bus stops. In formal terms, the transport network may be modelled using a directed graph *G* = (*N*, *A*), where *N* is the set of nodes, in which each node *p* Є *N* represents a point of the transport network. *A* is the set of directed arcs, wherein each arc *a* Є *A* represents the section of the route connecting two nodes *p*, *k* Є *N*, where *p* is the start node of the arc and *k* the end node of the arc. A route on the transport network is a route that runs systematically, starting at a node that is a stop on the route, the start node, and ending at another stop, the end node. The set of routes on the transport network is represented by *R* and each route on the network by *r*, *r* Є *R*. A route *r* is an ordered sequence of arcs such that *a* Є *A*, thus establishing the passing order for each node on the route: *r* = (*a*_1_, *a*_2_, …, *a*_n_). The subscript of each arc of the sequence indicates the position in the ordered sequence, such that the start node on the first arc of the sequence, *a*_1_, is the start node of the route and the end node of the last arc of the sequence, *a*_n_, is the end node of the route. Given a route *r*, defined by a sequence of *n* arcs, the number of nodes involved in the definition of a line will be *n* + 1, of which at least two must be a stop. To evaluate the punctuality of route *r*, stop nodes are required. This subset of nodes of route *r* will be called route *r* set of stops, and will be represented by *P_r_*; they are an ordered set of stop nodes, in which the order of each node in the set *P_r_* matches the order in which they are visited on route *r*. A public transport operator’s schedule, *SP*, consists of a set *SP* = {*S_i_*}, where each element *S_i_* is called a scheduling unit. Each scheduling unit, *S_i_*, is assigned a vehicle resource, a driver resource and a set of operations to be carried out at a scheduled date and time. If the schedule is not met, this means an inefficient use of the allocated resources. The operations included in a *S_i_* scheduling unit may be of different types, but for the purposes of monitoring quality of service, line service operations are of greatest interest. A line service is defined as the completion of route *r* at a scheduled date and time. The set of line service operations on route *r*, *L_r_* = {*l_i_*}, is the set that comprises all line service operations of route *r* established in *SP*; moreover, to represent only the line services on route *r* to be carried out during a period of time, *T*, the notation *L_r,T_* is used. For each line service, *l_i_*, passing times through each of the nodes of set *P_r_* are scheduled, and that will be the information advertised to transport users. On line service *l_i_* on route *r* with *n* stops, the timetabled passing times of said line service is an ordered cumulative sequence of times specifying what time, *t*, the vehicle is scheduled to pass each of the stops on the route, i.e., *TP_li_* = {*t*_1_, *t*_2_,…, *t*_n_} where *t*_1_ indicates at what time the vehicle is scheduled to start the line service, *t*_2_ the arrival time at the second stop, and so on until the last time, *t*_n_, which indicates the scheduled time at which the vehicle will reach the last stop on the route. The passing times of line service *l_i_* on route *r* vary and depend on various factors, some of which are related to transportation issues and some of which are not. More specifically, when scheduling passing times at the various stops on a line service, it is necessary to consider the time required to complete each of the arcs that define the route and the time needed by passengers to board or alight from the vehicle at each stop on the route. The time spent completing the different arcs of the route depends on the vehicle speed, which in turn depends on variable factors such as weather conditions when the line service is running or the traffic status, which in turn is a factor dependent on the time of day the route is operated or the day type, e.g., working day, weekend or holiday, and even on the time of year. To draw this paragraph on conceptual models to a close, we would like to mention that they are implemented through a database that contains all the entities and the relationships between the entities representing the transport network, the deployed resources and the records reflecting all the facets of public transport operations. This database is called the transport database (TDB).

As mentioned in the previous section on related studies, there are different criteria for assessing adherence to the timetabled passing times on the line services included in *SP*. To decide on what criteria to use, the type of line service must be taken into account. For line services scheduled by frequency, typical of urban transport, the criterion used is the regularity with which the service is provided. However, for line services scheduled by timetable, typical of intercity transport, the criterion used is punctuality. As was stated in the previous section, to evaluate the punctuality of the line service, the OTP and RTV metrics are used. RTV is calculated using a basic metric called Arrival Delay (AD) obtained for each stop, *p*, of a line service on route *r*, *AD_li,p_*. AD is defined as the difference between the observed passing time through stop *p*, *OT_li,p_*, and the scheduled passing time through that stop, *ST_li,p_*, when operating line service *l_i_*. Considering that the passing times for each stop are measured with respect to the time at which the line service began, this metric is calculated using the following expression:

(1) $$AD_{li,p}=OT_{li,p}-ST_{li,p}.$$

The RTV metric is calculated using the following expression:

(2) $$RTV_{li}=n^{-1}\times \overset{n}{\underset{p=0}{\sum }}\frac{\left|OT_{li,p}-ST_{li,p}\right|}{OT_{li,p}}$$

As stated in the previous section, for line services operating by frequency, the criterion used is regularity. To evaluate regularity, the HV and EWT metrics are used. To calculate these metrics, first the Headway Ratio (HR) parameter has to be calculated using the following expression:

(3) $$HR_{li,k}^{p}=\left(\frac{H_{li,k}^{p}}{f_{li,k}^{p}}\right)\times 100$$

where $f_{li,k}^{p}$ is thescheduled frequency between two line services *l_i_, l_i+1_* at stop *p* on route *r* and $H_{li,k}^{p}\mathrm{the}$ frequency observed between both line services at stop *p* on route *r*. The factor 100 represents a perfect match between observed and scheduled time. If for each pair of possible values for service lines *l_i_, l_i+1_*, the average and standard deviation of *HR* at each stop *p* on route *r* is calculated, then the HV metric is obtained as follows:

(4) $$HV^{p}=\frac{\sigma_{HR}^{p}}{\mu_{HR}^{p}}$$

The EWT metric is an estimate of excess user waiting time due to failure to meet the scheduled frequency of two line services on route *r*. Its value, at stop *p* on route *r*, is obtained from the value $HV^{p}$ using the following expression:

(5) $$EWT^{p}=\frac{{\left(\sigma_{HR}^{p}\right)}^{2}}{2x\mu_{HR}^{p}}$$

## 4. The Service Quality Control Problem

As discussed above, quality of service on regular passenger transport by road is evaluated by applying criteria which vary depending on whether line services are planned by frequency or by passing times. In the first case, the criterion used is regularity and the associated metrics to evaluate regularity are HV and EWT. In the case of line services planned by timetabled passing times, the criterion used is punctuality and the associated metrics are AD and RTV. From the mathematical expressions for calculating each of these metrics we may deduce that in order to obtain the values it is necessary to know the time at which the vehicle arrives and stops—when it stops to pick up or set down passengers—or the time at which the vehicle passes the stop—when it does not stop because there are no passengers to pick up or set down at the stop. To obtain these data it is necessary to know the position of the vehicle, and from this position to locate the vehicle on the transport network, an action performed by the vehicle’s positioning system. When locating the vehicle on the transport network, if the position is provided by GPS, it should be borne in mind that sometimes the location data are erroneous or may not be 100% accurate, depending on the data error caused by the various factors described in the previous section. When the vehicle passes a stop on the route without stopping, the time of passing is the moment at which it is nearest the stop; the distance between the stop and this point is defined by a preset threshold. By contrast, when the vehicle stops to pick up or drop off passengers, the arrival time at the stop is the moment at which the GPS data indicate that the vehicle is stationary and its distance in relation to the stop is below a defined threshold. When defining this threshold, the aforementioned GPS data error must be taken into account, as well as the variable length of the bus stop parking area; in the case of the transport network of this particular study this distance varied between 12 and 50 m. If vehicles only stopped at bus stops on their route, the arrival time at each stop could be obtained with a high degree of reliability, regardless of errors in GPS data. However, in reality no bus only stops at bus stops; they also stop because of traffic or because of the traffic signals along the route. The problem is how to distinguish the position of a bus that has stopped because of a traffic signal from the position of the same vehicle on the same route that has stopped at a bus stop when the position of the traffic signal and the stop are very close. This is a very common situation on urban bus routes.

Another challenge that must be addressed when studying the regularity or punctuality of line services on public transport is the reliability of the results. To achieve a high degree of reliability it is necessary to work with integral data sets, i.e., complete and correct sets of data. In the context of public transport, potential sources of error in the data are: failure in position data or in the devices that process the data, occasional errors due to noise or data that, although correct, have been obtained for routes that do not match the corresponding route plan. It is therefore necessary to carry out a filtering process to ensure data integrity when analysing the regularity or punctuality of line services.

A final challenge when conducting a comprehensive and ongoing evaluation of the punctuality or regularity of public transport services is the handling of the amount of data required, which in the case of medium to large transport networks is massive. Therefore, intelligent data handling is required.

## 5. System Overview

The proposed system is designed to continuously and comprehensively evaluate the quality of service provided by a regular road passenger transport operator. This evaluation is conducted using metrics commonly used by operators and transport agencies, measuring punctuality—e.g., with the AD and RTV parameters—or measuring regularity—e.g., with the HV or EWT parameters. Continuous evaluation of quality of service means that it may be performed at any given time, thus permitting its evolution to be analysed in different calendar periods (working days, holidays, weekends, time of year, etc.). Comprehensive evaluation means that the metrics can be obtained with different levels of granularity, i.e., for a set of routes, for specific routes, or for a set of individual stops.

**Figure 2:**
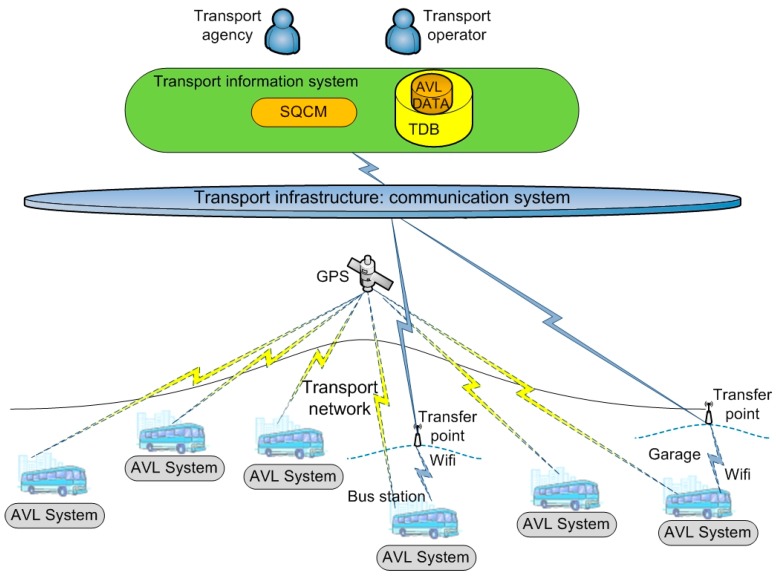
System Overview

The system primarily uses four resources that are usually employed in regular public passenger transport by road: GPS vehicle tracking system, the computing and storage systems used in vehicles to record and monitor the activities carried out on board, the communications system used to transfer data between the head office and the vehicles, and the transport database (TDB). The system consists of two modules: the AVL Module (AVLM) deployed on the vehicles and the Service Quality Control Module (SQCM) deployed at the operator’s or transport agency’s data processing centre. The AVLM continuously records and automatically transfers the vehicle’s position. The SQCM evaluates the quality of service by calculating the punctuality and regularity metrics for the planned services. Figure 2 provides an overview of the system.

### 5.1. The AVLM

The mission of this module is to obtain the basic data required to evaluate the punctuality or regularity of the line services performed by the vehicle. These data are obtained from the GPS vehicle tracking system. The data recorded are geodetic position, speed, data quality and the time at which the data were obtained. Table 2 shows the recording structure used to store each GPS location reading, specifying the meaning and size of each field. Henceforth these data will be referred to as RAVL.

The RAVL_Age_ and RAVL_Sou_ fields indicate the quality of the GPS data; data with a value of 2 in both fields indicates high quality, any data with a value of 0 in either of these fields indicate that the data are unreliable. A value of 1 in the Age field indicates that the data were obtained more than 10 s earlier. A value of 1 or 2 in the Source field indicates that it has been possible to obtain the latitude and longitude coordinates—i.e., two-dimensional location data—which occurs when the vehicle’s GPS receiver was unable to obtain the signal from at least four GPS satellites.

The AVL module is run on the onboard computer typically used by transport companies to record events during vehicle service. This computer has a display, keyboard, cable communication interfaces to connect up the other devices on the vehicle (card readers, vehicle information panels, sensors, etc.), and wireless communication interfaces for data communications: 3G for long-distance data communications and WiFi for local communications. To transfer location data this module uses WiFi infrastructure.

Because of the potentially massive number of RAVL records—as the position of each vehicle is recorded continuously—the limited storage capacity of the onboard computer and the limited bandwidth of the WiFi communications, the AVLM handles data intelligently to minimise the following aspects:

- The number of AVL records required for quality control.
- The time AVL records are stored on the vehicle.
- Errors in data communications.

To minimise the number of AVL records, this module uses a variable sampling period, which depends on the time unit used in planning timetables for each line service. For example, for long-distance line services the smallest unit of time is a minute. In addition, vehicle position sampling is only performed on a scheduled service, since when the vehicle is not in service or is performing an unplanned operation, no control is required.

To minimise the time that RAVL records are stored on the onboard computer, they may be transferred at different points of the transport network through which vehicles usually pass several times a day; for example, stations or garages.

To minimise errors in RAVL file transfers, caused by the fact that the vehicles are in motion and therefore WiFi connections are intermittent, the AVLM connects to WiFi by selecting the most suitable place and time using position and speed data provided by the vehicle’s GPS and timetable information. More precisely: transfers are only made at those parts of the transport network where wireless coverage is available and where the vehicle is scheduled to remain stationary for a sufficient period of time for data transfer to be completed. With the information from the vehicle tracking system, the AVLM knows when it is at a WiFi point, with the timetable information the AVLM knows how long it should remain at that point, and with the vehicle’s speed data provided by GPS, the AVML can initiate data transfer when the vehicle has stopped at that point, thus avoiding transmission errors.

### 5.2. The SQCM

**Figure 3:**
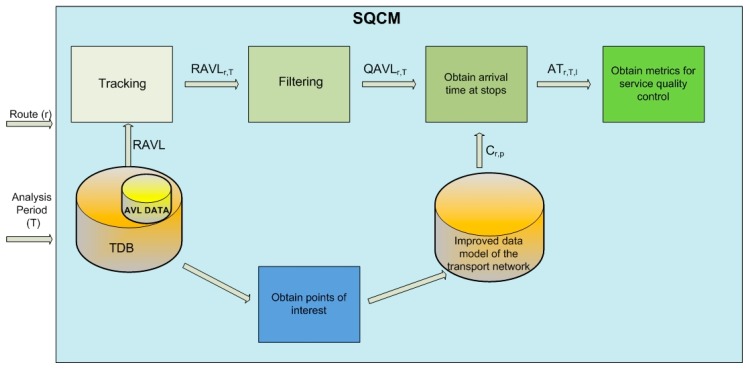
SQCM Phases.

Based on specification of the part of the transport network to be analysed—i.e., a stop, a route, a set of line services, etc.—and the temporal aspects of the analysis, this module obtains the data and parameters required for quality control. The SQCM, as shown in Figure 3, is executed in four steps: the first step, tracking, consists of obtaining the initial set of position data that will be used for quality control; the second step, filtering, ensures that quality control will be carried out using an integral data set, selecting reliable position data to that end; the aim of the third step is to obtain the passing times for each of the stops on the line services analysed; and finally, the fourth step consists of calculating the metrics used for quality control.

#### 5.2.1. The Improved Transport Network Model

**Figure 4:**
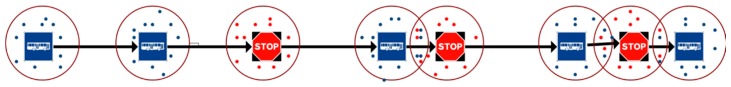
Cases of overlapping position data for stationary vehicles.

To calculate the various metrics used to evaluate the punctuality or regularity of a line service, the system must obtain the arrival time at each of the stops along the route. If the vehicles were only to stop at the bus stops, obtaining the arrival time at each stop would be simple. But in reality, the vehicles do not only stop at bus stops on the route, but also at unscheduled points due to traffic signs and congestion, specific incidents, etc. In order to distinguish when the position data for a stationary vehicle is due to the vehicle being at a scheduled stop or it having stopped due to traffic signs or congestion, the system must consider several scenarios. The least complex situation arises when a stop is at a distance greater than the maximum error in a GPS reading from the previous and next stops on the route, and also, the vehicle is not stopped due to a road sign or traffic conditions at a point on a radius greater than the maximum error of a GPS reading. The most complex situation arises when there is a stop and, next to it, at a distance less than the maximum error established by the system for GPS data, which was set at 25 m in the previous section, there is a traffic signal or a road feature—e.g., narrowing, roundabout or pedestrian crossing—which makes the vehicle stop. In this situation, the system must distinguish between GPS data for a stationary vehicle associated with it being at a bus stop, and the GPS data obtained when the vehicle was stationary due to traffic conditions or signs. In addition, it should be noted that because traffic conditions are variable, the points at which a vehicle might stop due to this factor are also variable. Figure 4 schematically represents cases of stationary vehicle data on a line service. The blue dots represent data for a vehicle when stationary at a stop. The red dots represent data for a vehicle that has stopped because of a traffic signal or congestion. The red circles represent the perimeter of radius *U* in which the vehicle is considered to be at a given point of the route. It is assumed that there can never be two stops at a distance below the threshold *U*. When a stop is near a traffic signal, there are areas in which stationary vehicle data are mixed owing to the vehicle being at a stop and because of the traffic signal.

In order to discriminate between the position data for stationary vehicles, our system uses an improved transport network representation model. The improvement consists in incorporating the points where vehicles stop systematically without being scheduled route stops. The technique used is based on a previous study published by the authors [32]. This technique uses the *k*-means classification method to classify all GPS stationary data obtained on the route under analysis and during the time interval in which quality control is being conducted. If the route has *n* stops, this classification process classifies all stationary data in *m* subsets, where *n* ≤ *m*. Of these *m* subsets, there will be *n* subsets of GPS stationary data associated with scheduled stops and the remaining subsets will be GPS stationary data associated with unscheduled stops. This classification process is described below. The rules used to identify the clusters are as follows:

- Cluster associated with a scheduled stop on the route. If the cluster has a centroid very close to a point in the GPS data set and the scatter of the readings belonging to the cluster is low, then it is a set of readings associated with a scheduled stop of the vehicle. Because each point represents an area reserved for the vehicle to stop, a centroid is considered to be very close to a point if the distance between them is not more than 25 m (maximum error). The first two sets of points in Figure 4 are cases of this type on the route being analyzed.
- Cluster associated with a traffic signal on the route. If the centroid of the cluster is not close to any bus stops and the scatter of the readings belonging to the cluster is low, then it is a set of readings associated with a singular point in the route, with the centroid being the position that represents the traffic signal being identified. The third set in Figure 4 is a case of this type along the sample route.
- Unidentified cluster. If the cluster exhibits high scatter in the GPS readings, then it is a cluster with readings associated with more than one point at which the vehicle stops systematically (bus stop or traffic signal). In Figure 4, the fourth and fifth sets illustrate a case of this type, just like the last three sets. For each cluster of this type, a new iteration is performed, and each time, the K-means algorithm restricted to the points assigned to the cluster being studied is applied, taking as an initial approximation in the second and following iterations the points associated with bus stops that are known, that belong to the cluster and the centroid of the cluster obtained. Thus, the points will be grouped on the new centroids, each of which is located around the bus stops and traffic signal points of the cluster. The operation must be reiterated until a result is obtained for which the points are grouped at short distances from the centroids of their cluster. If the centroid of one of these clusters is very close (less than 25 m) to a bus stop point known, then the cluster represents readings of bus stop. In contrast, if the centroid is not close to a bus stops point known then the cluster is associated with a point of traffic signal that has been identified.

The route to be analysed for quality of service is *r* and *P_r_* is the ordered set of *n* stops on the line. *RAVL_r,T_* is the set of GPS data obtained by all the vehicles that have completed route *r* during *T*, the period of time to be analysed. The subset *ZAVL_r,T_* is defined as the subset of *RAVL_r,T_*, consisting of only GPS stationary vehicle data. Thus:

$$\forall RAVL\in ZAVL_{r,T}:RAVL\in RAVL_{r,T}\wedge RAVL_{vel}=0$$

Once the *ZAVL_r,T_* set has been obtained, the first iteration of the *k*-means classifier is executed to split the *ZAVL_r,T_* set into *n* classes, taking as an initial approximation *n* centroids associated with each stop on the route. As a result *n* subsets of *ZAVL_r,T_* will be obtained, in which each subset *i* corresponds to the data belonging to class *i*, where 1 ≤ *i* ≤ *n*. Each subset *i* is represented by *ZAVL_r,T,i_* and its centroid by *C_r,T,i_*. If all *ZAVL_r,T,i_* clusters have only one stop and all GPS stationary data are at a distance from the centroid *C_r,T,i_* that is less than the threshold *U*, then the classification has converged in a solution in which we have reliably associated all stationary speed data with stops. Otherwise, if there is a cluster in which there is no stop, then that cluster contains GPS stationary data associated with unscheduled stops made by vehicles that have completed route *r* and there is at least one class with more than one stop. For each cluster with more than one stop the *k*-means classifier is only executed with the readings belonging to the class, and so on. Once all stationary data of a *ZAVL_r,T_* set have been classified, *m* classes are obtained, where *m* ≥ *n*. The number of *m* classes is greater than *n* when at least one class has been obtained with GPS stationary data to which no stop belongs, and these readings are due to the vehicle stopping systematically at a point which is not a bus stop; we shall call these points unscheduled stop points on route *r*. The centroids of this type of cluster are added to the geographic database of the transport network as points of interest, to be taken into account when calculating the journey times of the different routes that pass through these points. The set of *m* centroids associated with route *r* is represented by *C_r,T_*, and *C_r,T,p_* is a subset of this set and consists only of the centroids that represent the GPS stationary data associated with stops on the route. The subset *C_r,t,p_* will be used to obtain arrival times when vehicles stop at every stop *p*.

Below, Algorithm 1, we algorithmically describe the technique used to obtain the points of interest on route *r*, which as mentioned above are points where vehicles stop systematically without being stops on a route. The algorithm is recursive and uses three input parameters. The first parameter is a set of GPS coordinates for the route to be analysed, thus in the first invocation the *AVL_r,T_* set is used as the initial set of GPS data, consisting of the coordinates obtained by the vehicles when operating route *r* for time period *T*. The second parameter is the number of classes to be classified; in the first invocation the value of this parameter is the number of stops along the route. The third parameter corresponds to the initial approximations to each of the centroids of the classes; the input data in the first invocation are the GPS positions of the stops. As the output set, the *C_r,T_* set of centroids is obtained, representing each of the classes into which all the stationary data have been clustered.

**Algorithm 1. Stationary data Classifier**Procedure Classifier_ Stationary_Data (*Q_r_* , *N*, *C_r_*) **Input data:**    *Q_r_*: set of position data obtained by vehicles operating line services on route *r*.    *N*: number of classes to be classified.    *C_r_*: set of initial approximations to centroids. Output data:    *C_r_,_T_*: centroids representing each of the classes of GPS stationary data. Initial values: *Z_r_* = Ø; Step 1: Obtain *Z_r_* which is the set of GPS stationary data on route *r*: For each RAVL position reading belonging to *Q_r_*,  if *RAVL_vel_* = 0 then   include *RAVL* in *Z_r_*.  End if Done Step 2: Classifying by procedure *K*_means_classifier (*Z_r_*, *n, P_r_, Z_r,_ C_r_)* Input data:   *Z_r_*: *set* of stationary data to be classified.   *n*: number of stops on the route.   *P_r_*: positions of the *n* stops on route *r*. Output data:   *n Z_r_* clusters   *n C_r_* centroids of each cluster *K*_means_classifier (*Z_r_*, *n, P_r_, Z_r,_ C_r_ )*
 Step 3: Identifying the resulting clusters For each *Z_r,i_* resulting cluster, where 1 ≤ *i* ≤ *n*    If *Z_r,i_* cluster contains no stop then        Label the cluster as an unscheduled stop point.    End if    If *Z_r,i_* contains one and only one stop *p* on the line then        Select *Z_r,i_* as the set of readings to obtain the time of arrival at the stop on the line service of route *r*.    End if    If Zr,i contains k stops and k > 1 then:       Classifier_ Stationary_Data (*Z_r,i_*, *k*, *C_r,i_*)         Where:          *Z_r,i_*: the GPS stationary data belonging to class *i*.          *k*: number of stops in cluster *i*.          *C_r,i_*: positions of the *k* stops in *Z_r,i_*.    End if Done 

**Figure 5:**
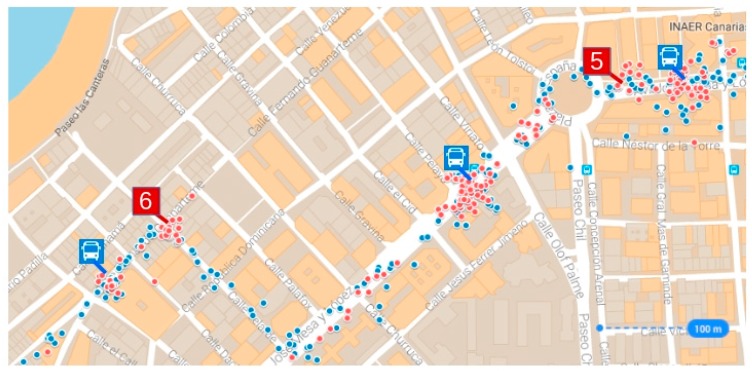
Example of a set of position data for vehicles in an urban section of the analysed route.

Figure 5 illustrates a real-life result for this classification technique applied to the route studied in this article. It shows a map of part of the route with the position data, represented by blue and red dots, and the positions of two centroids of stationary data clusters, the red icons labelled with numbers 5 and 6, acquired during the analysed line services. Three stops on the route are identified by blue bus icons. The red dots represent GPS readings with zero horizontal velocity used by the classification technique to identify centroids of stationary data clusters.

We can also see from the map that there is a section between two of these stops containing a roundabout, hence the classification technique applied to stationary data on this section produces three clusters; following the route, these correspond to the following: the first, to the first stop of the section, the second, to the point where vehicles stop to join the roundabout—the red icon labelled with the number 5—and the third, to the second stop. Therefore, to calculate the arrival time at each of these stops, only the data pertaining to each of their clusters are considered, and data pertaining to the clusters represented by the centroid labelled with the number 5 are ignored. A similar case occurs with the stationary vehicle points in the proximity of the point represented by the red icon labelled with the number 6, which corresponds to a point where there is a stop sign and to the bus stop closest to this point. The technique that we have described classifies these points into two clusters, using only stationary vehicle data belonging to the cluster associated with the stop to obtain the arrival time at this stop.

#### 5.2.2. Step 1: Tracking

The quality control process starts with specification of the route, *r*, and the period of time, *T*, during which it is to be conducted. From these initial specifications, the objective of this step is to obtain the RAVL set of data acquired when the line services on route *r* were completed in time period *T*, a set hereinafter represented by *RAVL_r,T_*. As the RAVL data are obtained periodically by all vehicles in the fleet while operating the line services, regardless of the service in question, *RAVL_r,T_* is a subset of the set that comprises all the RAVL data of the system. To select the RAVL data that are part of *RAVL_r,T_* it is necessary to first obtain the set of line services on route *r* that have been operated during the period in question, i.e., *L_r,T_*. For each line service *l_i_* Є *L_r,T_* we need to know the following: the vehicle that operated it and the time it began and ended. As we have already discussed in Section 3, all activity carried out by the transport operator is stored in the TDB. The data stored in this database include the start and end times of the line services, and this is where the data required for each *l_i_* Є *L_r,T_* are obtained. This TDB query produces a data set in which each record represents a *l_i_* Є *L_r,T_*; Table 3 shows the structure of each of these records.

- Once the required data from each *l_i_* Є *L_r,T_* have been obtained, the *RAVL* Є *AVL_r,T_* data will be those for which the vehicle field, *RAVL_Veh_*, coincides with the vehicle field of a *l* Є *L_r,T_* record and the time that the position was captured; *RAVL_T_* is greater than or equal to the time at which line service *l*, *l_T_*_0_ began, and less than the time at which line service *l*, *l_T_*_1_ ended. In formal terms:
  $$\forall RAVL\in AVL_{r,T},\exists !l\in L_{r,T}:RAVL_{Veh}=l_{Veh}\wedge l_{T0}\leq RAVL_{T}\leq l_{T1}$$
- For there to be a reliable relationship—based on time data—between the data from the TBD and the AVL module, both components must be synchronised using the same clock source. Therefore, the driver’s console uses the GPS receiver clock to synchronise its clock.

#### 5.2.3. Step 2: Filtering

For the quality control to be reliable, data integrity must be ensured; this is the purpose of this step in the process. Data integrity means that there are no erroneous data and that the dataset is complete. For our system, this integrity is achieved by executing filtering rules: the first, to eliminate from the *RAVL_r,T_* set those records containing position data that are not good quality; the second, to remove those records that are not part of a sequence of position readings that represent a complete and consistent route.

*RAVL_r,T_* records with low-quality GPS data are eliminated using the fields that indicate the age of the data, *RAVL_Age_*, and type of data, *RAVL_Sou_*; thus the RAVL record is deleted if either of the following two conditions are met:

- The data are old, i.e., *RAVL_Age_* = 1, or no information is available for the age of the data, i.e., *RAVL_Age_* = 0.
- Information on the type of data obtained (2D or 3D) is not available, i.e., *RAVL_Age_* = 0.

*RAVL_r,T_* records that are not part of a complete and consistent route *r* are eliminated by checking that the sequence of RAVL records obtained for each line service *l_i_* Є *L_r,T_* is complete, and that it represents a route consistent with route *r*, i.e., it passes through all the stops and in the planned order. In formal terms:

- The line service is *l* Є *L_r,T_*, and *AVL_r,T,l_* is the set of position data obtained during line service *l*, where *l_T_*_0_ and *l_T_*_1_ are the start and end times of line service *l*, respectively, and *Q* the sampling period used to record the RAVL data during operation of the service. The data sequence *RAVL_r,T,l_* is then complete if the number of records in this sequence, *NAVL_r,T,l_* is:
  $$NAVL_{r,T,l}=\frac{\left(l_{T1}-l_{T0}\right)}{Q}$$
- *C_r,T,p_* = {*c*_0_, *c*_1_,…, *c*_n_} is the ordered set of centroids associated with each of the stops along route *r*. The route represented by *RAVL_r,T,l_* data is thus consistent with route *r* if:
  $$\forall c_{i}\exists !RAVL:Dist\left(RAVL_{p},c_{i}\right)\leq U,$$
  where *Dist* is the distance between two points, *RAV_Lp_* the position logged in a *RAVL* Є *RAVL_r,T,l_* record, and *U* a distance threshold representing proximity. This value depends on the maximum GPS error accepted by the system, *E*, the length of the parking space at the bus stop, the maximum speed that the vehicle may attain and the time period used by the vehicles to acquire their GPS position data.

As a result of this step, an integral set of RAVL records is obtained, i.e., data containing position data of good quality and representative of line services on route *r* that are complete and consistent with route *r* as planned. Hereinafter, this set will be represented by *QAVL_r,T_*. Similarly, the set of reliable data obtained during a line service *l*, on route *r* and operated during the period *T* will be represented by *QAVL_r,T,l_*.

#### 5.2.4. Step 3: Obtaining Passing Times

Once the *QAVL_r,T_* set has been obtained, the next step is to obtain the position data captured at the time that the vehicle arrives at each of the stops. To this end, the system partitions the *QAVL_r,T_* set into subsets of *QAVL_r,T,l_* data. If the position data for each of these *QAVL_r,T,l_* subsets are ordered on an ascending scale according to the time that each record was obtained, we would have a representation of a complete and consistent route *r* operated by the vehicle in question. The passing times for each of the stops on each of these *QAVL_r,T,l_* routes, which will be represented by the ordered time sequence *AT_r,T,l_*, will be obtained considering two different cases:

- Case A: the vehicle passes stop *p* and does not stop. This case occurs when there are no passengers waiting to board the vehicle at *p*, and no passengers on the bus who want to alight at *p*. In this case, the passing time for this stop is obtained by finding the position for the *QAVL_r,T,l_* set that is at a minimum distance from the position of stop *p*. The distance threshold used in this case is the maximum error that the system assumes for a GPS reading, the variable *E* which was fixed at 25 m in Section 3.
- Case B: the vehicle stops at stop *p*. This case occurs when there are passengers at the stop or passengers on the bus who want to alight at *p*. In this case, the time of arrival at stop *p* is obtained by taking centroid *C_r,p_*—obtained in the classification of GPS stationary vehicle data for route *r* as described in Section 5.2.1—as the stop position. The arrival time at stop *p* of a service line *l* on route *r* is the time at which the GPS position for the *QAVL_r,T,l_* set was recorded, and which must meet the following conditions:
  ○ It is stationary.
  ○ Of all the *QAVL_r,T,l_* stationary data, it is the nearest to *C_r,p_*.

#### 5.2.5. Step 4: Calculating the Metrics

Having obtaining the passing times for each of the stops on line service *AT_r,T,l_*, the values of the various metrics used to evaluate the punctuality or regularity of the service line are then obtained. To monitor punctuality, for line services scheduled by timetable, the basic parameter is *AD* (1). From this value the remaining metrics used to evaluate punctuality may be obtained. To monitor regularity, for services scheduled by frequency, the basic parameter is *HR* (3). From this value the remaining metrics used to evaluate regularity may be obtained.

## 6. Use Case: Results and Discussion

**Figure 6:**
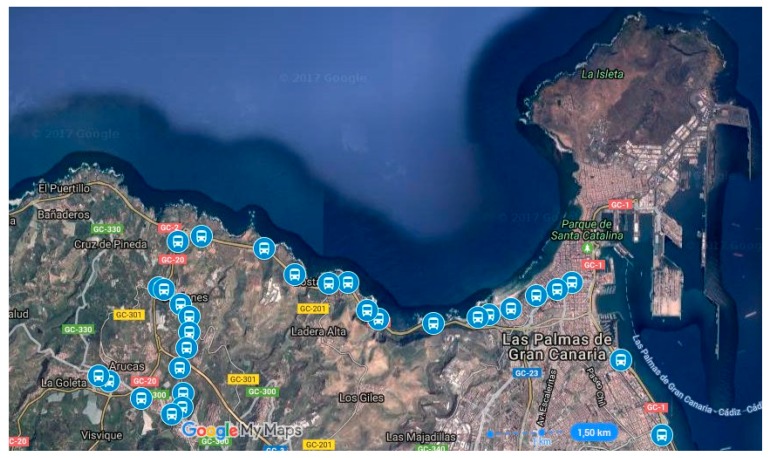
Aerial view of the route used for the case study. The bus icons represent stops on the route.

Currently the system described in this article is in operation at the public transport company, Global Salcai-Utinsa S.A. This company is based on the island of Gran Canaria (Canary Islands, Spain), has a fleet of 345 vehicles, and operates 127 different routes on a transport network that contains 2686 stops. In 2015, this company carried out an average of 2395 line services on all routes, covering 28,897,002 km and carrying 19,284,378 passengers in that year. The system has been integrated with the other components used by this company on its onboard systems and its communications systems. The quality control results obtained for one of its routes are described below. The route itself is 23 km long, starts in the city of Las Palmas and ends in the city of Arucas; it has 30 stops, and it begins in an urban area in the city of Las Palmas, until the sixth stop, after which it becomes an intercity route, until the last four stops, where it again becomes an urban route through the town of Arucas. The company uses the code 210 to identify the route; therefore in the formalisation developed for this article, *r* = 210. Figure 6 gives an aerial view of the route with the stops represented by bus icons.

**Figure 7:**
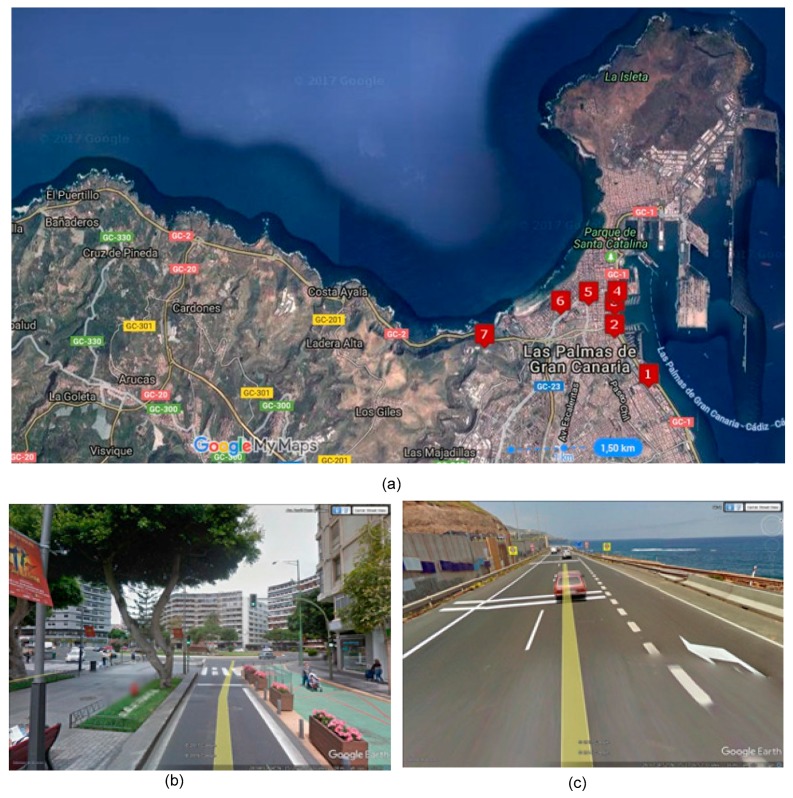
(**a**): aerial photo showing the location of the 7 unique points on the route. (**b**): photograph of point 5. (**c**): photograph of point 7.

The results were obtained during period *T*—the whole of 2015—therefore in our formalisation it will be indicated as follows: *T* = 2015. Route 210 was scheduled by timetable during the analysed period. The AVL system recorded 51,499,404 position readings during all the line services completed for this route. The number of vehicles involved in these line services for the seleted route was 166, being 16 the number of vehicles that carried out these services systematically. In the preliminary analysis of the route to detect systematic stopping points that are not stops on the route, 7 such points were identified. Figure 7a displays these points as numbered red icons. These points are the centroids of a set of stationary vehicle data. Figure 7b,c are photographs of two of these points. Figure 7b corresponds to point number 5; this is a systematic stop point because the road on which the vehicle is travelling is a bus lane ending in a roundabout, and entry into the roundabout is controlled by a traffic light. Figure 7c is a photograph of point number 7; this is a point where the vehicle stops because it is on a slip road (right lane) that joins a dual carriageway (left lanes) and does not have right-of-way.

The tracking phase was applied to this data set, and 617,195 position records were selected for 9675 line services on route 210 in 2015; these data form the set *AVL*_210,2015_. The filtering phase was applied to this set of GPS data, and verified 8572 line services for which the recorded data sequences represented complete and consistent routes; hence the set *QAVL*_210,2015_ contained 222,382 position records.

**Figure 8:**
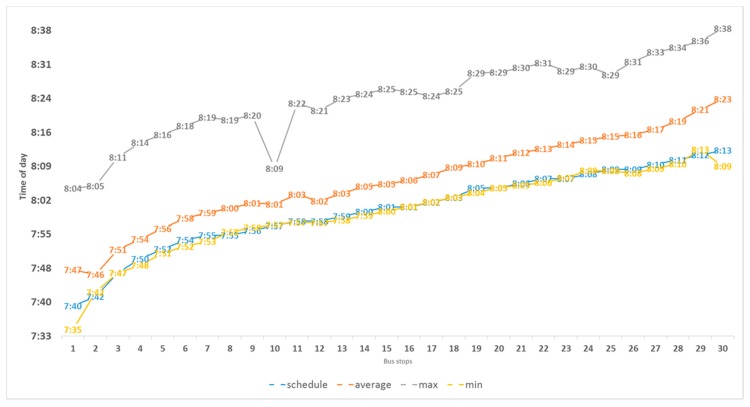
Passing times of line services starting at 07:40 Monday to Friday, excluding public holidays.

Once the GPS data had been filtered, we obtained the arrival times at each stop on each line service. The timetables of the line services on this route are scheduled by passing time, with two day types: one the one hand, weekdays (Monday to Friday), and on the other hand, weekends and public holidays. The results correspond to the two types: first, the results for weekday line services starting at 07:40 a.m., and then the earliest Sunday service, which starts at 8:40 a.m. Figure 8 shows the arrival times at each stop for the first set of line services, those that run from Monday to Friday. The horizontal axis represents the stops in order, i.e., the value 1 is associated with the first stop on the route, 2 with the second and so on until the last stop of the route, which has the value 30. The blue line represents the scheduled passing time, the average passing time is represented by the orange line, the earliest passing time by the yellow line, and the latest passing time by the grey line.

**Figure 9:**
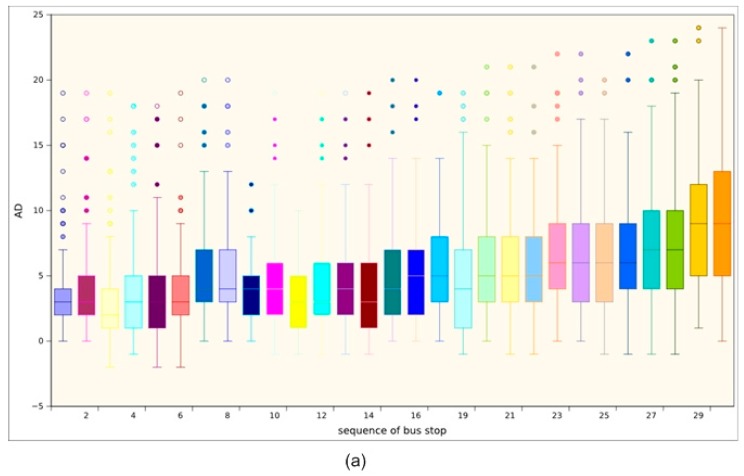

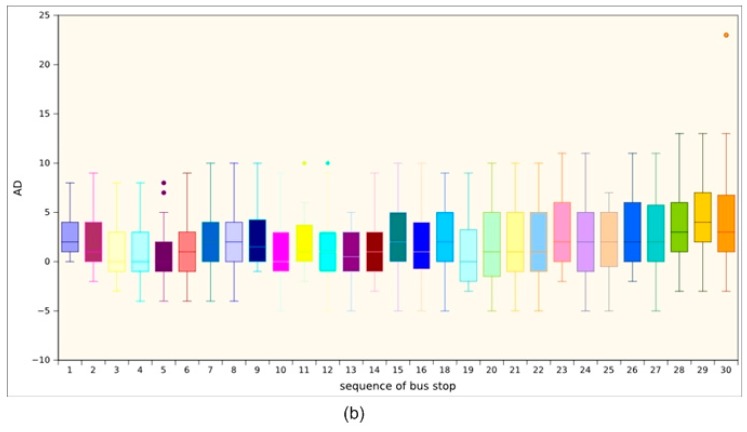
Box-and-whisker plots showing median values for the AD metric at each of the stops on the route for line services that run from 07:40 from Monday to Friday (plot (**a**)) and from 08:40 on Sunday (plot (**b**)).

Figure 9 shows two box-and-whisker plots displaying the percentile distribution of the AD metric at each stop on the route for line services that start at 07:40 from Monday to Friday (plot (a)) and for each stop on all line services that start at 08:40 on Sunday (plot (b)). In the plots, the value 0 on the vertical axis represents perfect timetable adherence, each band inside the boxes represents the median value, i.e., the delay value below which 50% of the AD values are located. The circles represent outliers: very small, very large or very rare values.

**Figure 10:**
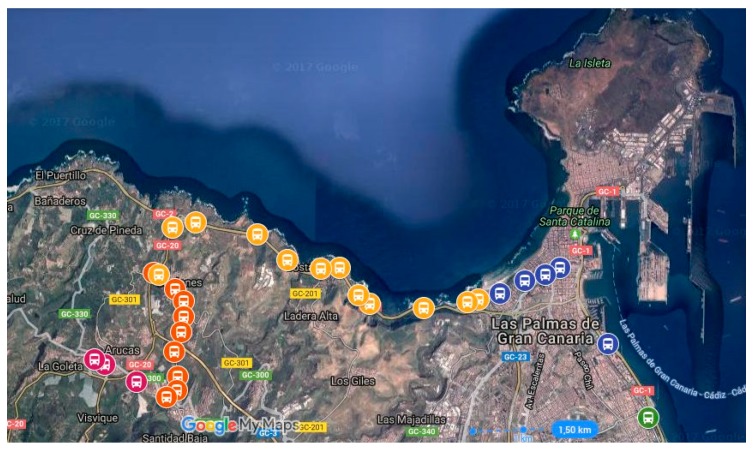
Stop categories according to delay, irrespective of day and time.

As can be seen, the line services on Sundays are much more punctual than those that run on weekdays, possibly due to the difference in traffic congestion between weekdays and Sundays. We may also note that, for weekday line services and for most of the stops, delays continue or increase the further along the route the bus is, ranging from 2 to 8 min. By contrast, on Sunday line services, the increase and decrease in delays at stops does not follow a fixed pattern, and the median value is maintained between 0 and 5 min.The AD parameter values for each stop of the line service on the route are displayed in Figure 10, irrespective of day type and time. The median values for delay times are represented by colours: the green stops indicate a median value of less than 1 min, blue stops a median value between 2 and 3 min, light orange stops between 4 and 5 min, dark orange stops between 6 and 7 min and, finally, the red stops represent a median delay value of more than seven minutes.

**Figure 11:**
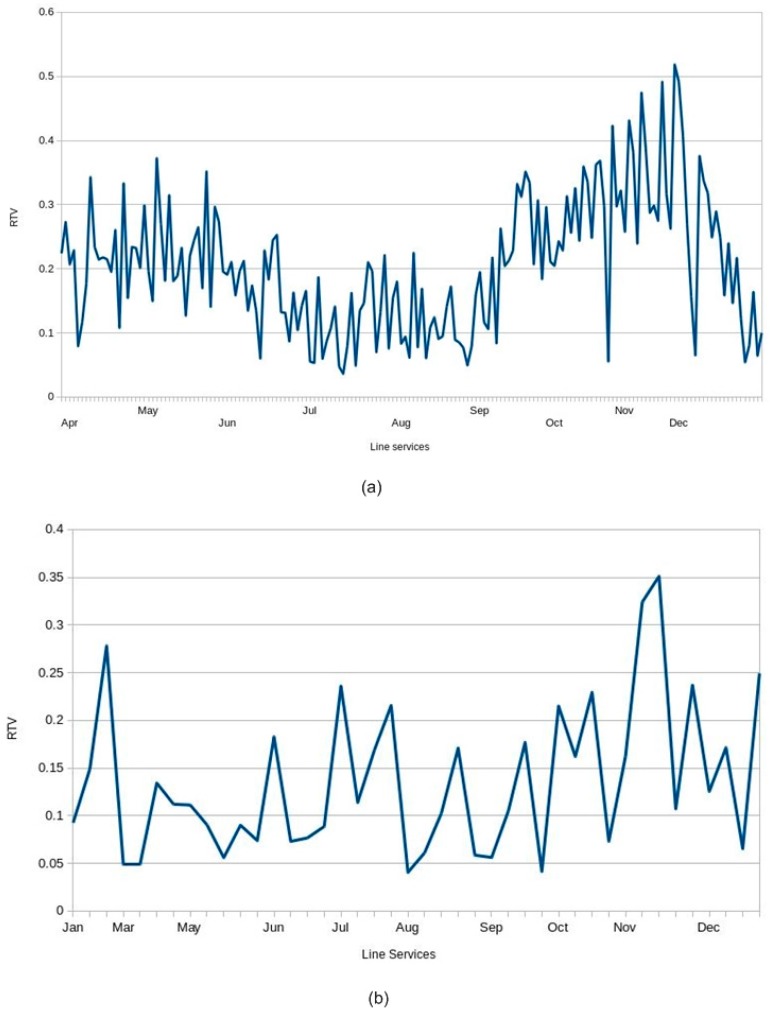
RTV metric values obtained during 2015. Graph (**a**) displays the values for each weekday service line that runs from 07:40. Graph (**b**) displays the values for each service line that runs on Sunday from 08:40.

Figure 11 shows the RTV value for each type of line service. Graph (a) shows the values for weekday services (Monday to Friday). Graph (b) shows the values obtained for Sundays. Comparing the figures, it may be seen that punctuality varies according to the day type: the line service is less punctual on weekdays than it is on Sundays. The explanation for this is that there is less traffic on Sunday and there is lower demand at that time, so the buses run more smoothly and quickly, and are thus more punctual. We may also note that during the months of July and August, the weekday services are more punctual than during the rest of the year, possibly because those months are typically during the main holiday period, so there is less traffic and lower demand.

The system described in this article provides two main contributions. The first is that it is a complete, detailed and realistic description of a system for measuring the regularity and punctuality of regular public passenger transport by road, solving the challenge of massive data management involved in continuously monitoring regularity and punctuality. The description is complete and detailed because it includes all the formal and technological components and details of the system. It has been implemented with elements that are commonly used by transport operators, thereby facilitating implementation and deployment. The second contribution is the technique used to obtain the arrival times at the stops of the transport network. This technique is the *k*-means classification method, used to distinguish stationary data due to scheduled stops from stationary data due to the existence of an unscheduled stop, i.e., due to a traffic sign or traffic congestion or the condition of the road on which the vehicle is travelling.

## Figures and Tables

**Table 1:** A selection of GPS performance metrics.

Metric	Value
SIS URE Accuracy	≤7.8 m 95% Global average URE during normal operations over all AODs
	≤6.0 m 95% Global average URE during normal operations at zero AOD
	≤12.8 m 95% Global average URE during normal operations at any AOD
	≤30 m 99.94% Global average URE during normal operations
	≤30 m 99.79% Worst-case single point average URE during normal operations
Position Service Availability	≥99% Horizontal, average location
	≥99% Vertical, average location
	≥90% Horizontal, worst-case location
	≥90% Vertical, worst-case location
Position Accuracy	≤9 m 95% Horizontal, global average
	≤15 m 95% Vertical, global average
	≤17 m 95% Horizontal, worst site
	≤37 m 95% Vertical, worst site

**Table 2:** Description of the RAVL data structure.

Field	Description (Size)
RAVL_Veh_	Vehicle identifier. (2 bytes)
RAVL_T_	GPS Time of day expressed in Coordinated Universal Time (UTC). (4 bytes)
RAVL_Lat_	Latitude of the vehicle expressed in degrees. (4 bytes)
RAVL_Long_	Longitude of the vehicle expressed in degrees. (4 bytes)
RAVL_Alt_	Altitude of the vehicle position expressed in feet. (4 bytes)
RAVL_Vel_	Horizontal speed of the vehicle expressed in miles per hour. (2 bytes)
RAVL_Age_	Age of the GPS data: 0: data not available 1: >10 s 2: <10 s (1 byte)
RAVL_Sou_	Type of position measurement: 0: data not available 1: 2D GPS 2: 3D GPS (1 byte)

**Table 3:** Structure of the table of records obtained from the TDB query.

Field	Description
*l_veh_*	Vehicle identifier
*l_T_*_0_	Time that the line service on route *r* began, expressed in UTC.
*l_T_*_1_	Time that the line service on route *r* ended, expressed in UTC.
